# Development of a method for detection and quantification of *B. brongniartii* and *B. bassiana* in soil

**DOI:** 10.1038/srep22933

**Published:** 2016-03-15

**Authors:** L. Canfora, E. Malusà, C. Tkaczuk, M. Tartanus, B.H. Łabanowska, F. Pinzari

**Affiliations:** 1 Council for Agricultural Research and Economics - Research Centre for the Soil-Plant System, Via della Navicella 2-4, 00184 Rome, Italy; 2 Research Institute of Horticulture, ul. Konstytucji 3 Maja 1/3, 96-100 Skierniewice, Poland; 3 Department of Plant Protection and Breeding, Siedlce University of Natural Sciences and Humanities, Prusa 14 Street, 08-110 Siedlce, Poland; 4 Life Science Department, Natural History Museum, Cromwell Road, London, SW7 5BD, UK.

## Abstract

A culture independent method based on qPCR was developed for the detection and quantification of two fungal inoculants in soil. The aim was to adapt a genotyping approach based on SSR (Simple Sequence Repeat) marker to a discriminating tracing of two different species of bioinoculants in soil, after their in-field release. Two entomopathogenic fungi, *Beauveria bassiana* and *B. brongniartii,* were traced and quantified in soil samples obtained from field trials. These two fungal species were used as biological agents in Poland to control *Melolontha melolontha* (European cockchafer), whose larvae live in soil menacing horticultural crops. Specificity of SSR markers was verified using controls consisting of: i) soil samples containing fungal spores of *B. bassiana* and *B. brongniartii* in known dilutions; ii) the DNA of the fungal microorganisms; iii) soil samples singly inoculated with each fungus species. An initial evaluation of the protocol was performed with analyses of soil DNA and mycelial DNA. Further, the simultaneous detection and quantification of *B. bassiana* and *B. brongniartii* in soil was achieved in field samples after application of the bio-inoculants. The protocol can be considered as a relatively low cost solution for the detection, identification and traceability of fungal bio-inoculants in soil.

In the last decade the application of biological control agents (BCA) and biofertilizers has started to be considered a feasible and effective alternative for pest control or to improve plant nutrition efficacy^1^,^2^,^3^. Different microorganisms, belonging to several species of bacteria and fungi, started to be used as inoculants^4^. However, despite the huge amount of studies and findings on beneficial strains, the application of microbial-based fertilizers and BCAs in agricultural practice is still hindered by several factors. These include the standardization of the products, whose quality can affect the inoculant viability and persistence in soil, as well as the possibility of tracing the inoculant in the environment^5^,^6^.

The assessment of inoculants persistence and traceability in soil is a key task to improve their efficacy, since it is the basis for understanding their behaviour in the crop environment, and to fine-tune their application method^7^,^8^. Verifying and monitoring their presence can thus provide knowledge of the functioning and robustness of the bio-inoculant itself. So far, the fate and persistence of target microorganisms in environmental samples has been assessed with a variety of culture-dependent and independent methods^9^,^10^,^11^,^12^,^13^,^14^,^15^. Among the various approaches used for tracing and quantifying mycorrhizal and pathogenic fungi in the environment, PCR-based methods have been the most used to address some of the key issues in the assessment of the microorganism persistence in soil^16^,^17^,^18^,^19^,^20^. These key issues regard also the monitoring methods that can properly identify the inoculated strains, distinguishing them from the native populations resident in soil.

There are several DNA fingerprinting methods used to probe inoculated strain/s, but they are mainly qualitative and not quantitative^19^,^20^,^21^,^22^,^23^,^24^. Culture-independent DNA-based detection of strains directly in soil-extracted DNA could reduce the time and costs of monitoring the persistence and amount of bio-inoculants in soil after their application. Among the available culture-independent methods, different approaches can be used to investigate soil microbial communities^25^,^26^,^27^,^28^. Such analyses have been performed based on universal primers, which, however, have not yet been used to discriminate between non-native and native soil microorganisms, nor between two different bio-inoculants.

To overcome such technical gaps, we have developed a culture-independent method based on simple sequence repeat (SSR or microsatellites) markers and qPCR for the direct, discriminant and simultaneous detection and quantification of two bio-inoculants in soil. Multilocus simple SSR genotyping is a technique used in the characterization of cultivated fungi based on PCR amplification of multiple loci^29^. Several fungal SSR markers have been reported to be species specific, with a polymorphic character, thus producing a highly discriminant fingerprint^22^,^30^. The qPCR with probes targeting genes of interest allows to quantify their copy number, providing information on the relative abundance within the microbial community^31^, so it could be used to monitor the dynamic of the presence of the specific inoculant after its application in the field. The aim of our study was to adapt a genotyping approach based on SSR markers to the discriminating tracing of two different species of bio-inoculants in soil, after their field application as biocontrol agents. The proposed method was tested for tracing and quantifying in soil two entomopathogenic fungi, *Beauveria bassiana* (Bals.-Criv.) Vuill. and *B. brongniartii* (Sacc.) Petch. used as BCAs of *Melolontha melolontha* L. (European cockchafer) in field trials.

## Materials and Methods

### Fungal strains and preparation of the bio-inocula

The strain of *B. brongniartii* was isolated on a selective medium^32^ from the soil of a potato field highly infested by *M. melolontha*. The soil samples were collected in May 2012 in Romanów locality (Lublin voivodship, Eastern Poland). The strain was deposited in the Fungal Collection of the Department of Plant Protection and Breeding, Siedlce University of Natural Sciences and Humanities. The sequence has been deposited in the GenBank database and can be accessed to ID KT932309.

The strain of *B. bassiana* was selected from rhizospheric soil of an apple orchard located in Valle d’Aosta by the company CCS Aosta, (Aosta, Italy) and named BB59. The sequence has been deposited in the GenBank database and can be accessed to ID KT932307.

Both bio-inocula were grown in a liquid medium based on malt extract and glucose and formulated as a wettable powder into a carrier material made of a mixture of corn fibres and zeolite (1:10 w-w). The concentration of each of the two fungi in the inoculum was about 1 · 10^7^ CFU · g^−1^.

### Field application of the *B. brongniartii* and *B. bassiana* strains and soil sampling

Two experiments were established on different field plots, each designed with 3 replications in a split plot design, located near Brzostówka (Lublin, Poland). Both fields (about 1 ha) were highly infested by *M. melolontha* larvae. The first trial (A) was carried out on an established strawberry plantation. The second trial (B) was on a bare field, on which, testing an integrated effort to control the white grubs of the cockchafer, buckwheat was previously grown in spring for green plowing (June) and soil disinfection was carried out (at the beginning of August) on half of the plots by active steaming^33^,^34^,^35^.

The inocula of *B. bassiana* or *B. brongniartii* were applied as an aqueous suspension, after overnight incubation of the bio-inocula in water containing about 10% of a stillage from yeast production (organic fertilizer) to foster the fungal growth. Each inoculum was applied singly at a dose of 15 kg·ha^−1^ to the soil, splitted into two applications with three weeks interval. When the two species were applied together, 7.5 kg·ha^−1^ of each inoculum was distributed with the same application schedule. In case of the strawberry plantation (trial A), the applications were carried out in June, near the plants row, with a sprayer having nozzles of large diameter, without a fan, to reduce the risk of damage of the fungal cells. In case of the bare field trial (B) the bio-inocula were distributed the day after the soil disinfection treatment with active steam and again after three weeks. After each application, the soil was mixed on the surface with a light hand hoeing.

Soil samples were collected three weeks after the last application. Ten sub-samples of about 50 g each were gathered from each plot and then they were mixed to obtain a composite sample used for the DNA analyses. They were collected either in the vicinity of the plant roots or in the inter-row. Soil samples were also collected from non-inoculated parts of the field as a negative control. However, since other strains of *B. brongniartii* or *B. bassiana* could naturally occur in the soil considered as negative control, the proposed method should have been able to trace both these and the inoculated strains.

### Extraction of genomic soil DNA

DNA was extracted in duplicate from 0.6 g of three bulk soil (from ten sub-samples) using the PowerSoil DNA Isolation Kit (Mo Bio Laboratories, Carlsbad, CA) according to the manufacturer’s protocol. Duplicates were then pooled for downstream analyses. Nucleic acids were eluted in 100 μl of elution buffer (MoBio). The concentration of DNA crude extracts was checked by Qubit^®^ 2.0 Fluorometer following manufacturer’s instructions kit and stored to −20 °C for PCR analysis.

### DNA isolation of Beauveria brongniartii and B. bassiana strains

*B. brongniartii* and *B. bassiana* strains were cultured on fresh PDA agar (Oxoid S.p.A., Milan, Italy) for 10 days at 25 °C. Mycelium and spores were harvested aseptically from each culture using a sterile scalpel. The fungal biomass used for DNA isolation was harvested from about 1 cm^2^ of agar surface, and was approximately the same for each of the two strains. Genomic DNA was extracted from 0.5 g fresh mycelium by using PowerSoil DNA Isolation Kit (Mo Bio Laboratories, Carlsbad, CA) as described above, and quantified using Qubit^®^ 2.0 Fluorometer kit following manufacturer’s instructions.

### Discriminant and quantitative analysis of SSR markers in soil DNA

Three *B. brongniartii* and three *B. bassiana* SSR primers pairs were tested for species specificity and performance in soil DNA extracts and in mycelia DNA extracts. For *B. brongniartii* SSR marker detection the following primers were tested: i) Bb1F4, Bb2A3, Bb4H9^22^. The three primers were evaluated according to the method of Enkerli *et al.*^22^ and were used to amplify *B. brongniartii* and *B. bassiana* genomic DNA, and soil DNA samples, in order to test the specificity and the traceability of *B. brongniartii* in soil.

For the detection of *B. bassiana* SSR marker, the following primers were tested: i) Ba01, Ba02, Ba03^30^. The three loci were selected according to the method of Rehner and Buckley^30^, which was applied to both fungal genomic DNAs, to the DNA extracted from the soils investigated in our experiments, and to a selection of soil fungal isolates, in order to test the specificity and the traceability of *B. bassiana* in soil. However, the specificity of these SSR primers was already broadly confirmed in the above mentioned studies by Enkerli *et al.*^22^ and Rehner and Buckley^30^. Both sensitivity and specificity in discriminating the two *Beauveria* species by the chosen SSR loci were thus tested by PCR and checked on 2% agarose gel with ethidium bromide staining^36^. The method was then adapted to qPCR Real Time conditions.

A calibration towards the sensitivity of the applied protocol was performed using liquid dilutions of fungal spores’ suspension with a known concentration of spores. The suspensions were prepared from *B. brongniartii* and *B. bassiana* cultures grown on Potato Dextrose Agar (Oxoid S.p.A., Milan, Italy), after a 10-day incubation at 25 °C, aseptically harvesting the spores from the surface of the colonies, by a sterile cotton swab, and suspending them in a three-fold diluted Czapek broth (Oxoid S.p.A., Milan, Italy). Spores’ concentrationwas measured in each suspension by counting them with a hemacytometer (Thoma Chamber) according to Smith *et al.*^37^. The initial concentrations in the two fungal samples were: 26.5 · 10^4^ and 27.25 · 10^4^ spores · ml^−1^ for *B. brongniartii* and *B. bassiana,* respectively. Four sequential dilutions in sterile distilled water of the initial suspensions were then prepared (from 10^4^ to 10^1^ spores · ml^−1^).

The dilutions were used as such and also added to soil samples. The soil used as basis for obtaining the microcosms with a known number of fungal spores was a control soil, not previously treated in the field with the fungal inocula. One ml of each spores’ dilutions was added to 0.6 g of soil and gently mixed, in order to obtain soil microcosms with a known, decreasing, concentration of fungal spores from the two fungal strains. The microcosms obtained were thus used to establish a kind of standard curve in order to compare the efficiency, sensitivity and specificity of the SSR genotyping approach for fungal bio-inoculants traceability in soil.

DNA was extracted from the microcosms within 48 h after the addition of the fungal spores, in duplicate as described above. The concentration of DNA crude extracts was checked by Qubit^®^ 2.0 Fluorometer kit, following manufacturer’s instructions, and stored at −20 °C for further PCR analysis.

Standard curves were created, for each targeted gene, using soil samples containing a known concentration of fungal spores. The qPCR reactions were performed in duplicate within each DNA template.

PCR products were purified after the qPCR reaction with a Qiaquick PCR purification kit (Qiagen Inc., Chatsworth, CA, USA), then quantified by Qubit^®^ 2.0 Fluorometer kit, following manufacturer’s instructions and diluted in order to minimize the PCR bias^26^. The gene copy number was calculated using the following equation (http://scienceprimer.com/copy-number-calculator-for-realtime-pcr):


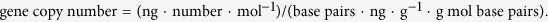


The standards were created using triplicate 10-fold dilution series covering six orders of magnitude from 10^2^ to 10^8^ gene copies per qPCR reaction during each run. *B. brongniartii* and *B. bassiana* SSR copy numbers were expressed per g^−1^ soil (dry weight).

All qPCR reactions were carried out in 25 μl reactions containing 10 μl of template DNA (2ng/μl), 12.5μl of Quanti Fast Sybr Green PCR Master Mix (Qiagen), 1.2 μM of primer, and PCR-grade water up to 15 μl. The reactions were performed in a Stratagene Mx3000P qPCR (Agilent Technologies). In order to protect soil DNA and mycelial DNA extracts from potentially present PCR-inhibitory substances, bovine serum albumin (BSA) was added to the SYBR green mix (Qiagen). Experiments were performed in duplicate. The results were processed using the program of the instrument Stratagene Mx3000P qPCR (Agilent Technologies). The absence of primers dimers in amplification products was evaluated analysing the melting curves of the products considering the fluorescence range 50–99 °C. Moreover, qPCR products were screened for purity and molecular weight in 1% agarose gel. Cycling condition was optimized running the qPCR at a different number of cycles, and selecting the protocol resulting in the best curves. Selected cycling conditions consisted of a 10 min initial denaturation at 95 °C and forty-two PCR cycles of 1 min at 95 °C, 40 s at 58 °C, and 30 s at 72 °C. The template quantities in soil samples were also compared with the template quantities in mycelia samples.

### Specificity of SSR markers

The fungal strains used in this study to further test the specificity of SSR markers are listed in Table 1. All fungal isolates were grown on potato dextrose agar (PDA, Oxoid S.p.A., Milan, Italy) at 25 °C. Genomic DNA was extracted from 0.5 g fresh mycelium by using PowerSoil DNA Isolation Kit (Mo Bio Laboratories, Carlsbad, CA) as described above, and quantified using Qubit^®^ 2.0 Fluorometer kit following manufacturer’s instructions. The amplification of fungal DNA was performed with the primers ITS1/ITS4, and the conditions used in PCR reactions were as follows: initial denaturation at 95 °C for 5 minutes; 34 cycles with denaturation at 94 °C for 1 minute, annealing at 60 °C for 1 minute, extension at 72 °C for 2 minutes; and a final extension of 8 minutes at 72 °C.

The quality of amplification products was checked by 1.5% agarose gels and ethidium bromide staining. PCR products resulting from three replicate reactions were pooled and purified with a Qiaquick PCR purification kit (Qiagen Inc. Chatsworth, CA, USA). Purified products were quantified by Qubit^®^ 2.0 Fluorometer following manufacturer’s instructions kit. Specificity was tested by PCR following the instructions reported above in the section 2.5. SSR marker detection for the locus Bb4H9 was performed according to the method of Enkerli *et al.*^22^ and was applied to all selected species (Table 1). SSR marker detection for the locus Ba01 was performed according Rehner S. A., and Buckley E. P.^30^.

### Statistical analyses

Data of qPCR quantification of DNA were obtained from soil samples where *B. brongniartii* and *B. bassiana* spores were added. A control consisting of soil without the inoculums was also included. The data were expressed as numbers of cells g^−1^ dry weight soil. Separated values of average number of copies of genes· g^−1^ dry soil are presented. qPCR results were analysed using one way ANOVA (*α* = 0.05), and the means within each treatment were compared for statistical significance of the differences by using Fisher’s Least Significant Difference (LSD) test at a significance level for p ≤ 0.05.

Data obtained from qPCR runs were plotted and compared using Origin 6.0 (Microcal, OriginLab Northampton, MA 01060 USA) software.

## Results

### Specificity of *B. brongniartii* and *B. bassiana* SSR markers detection

**Figure 1:**
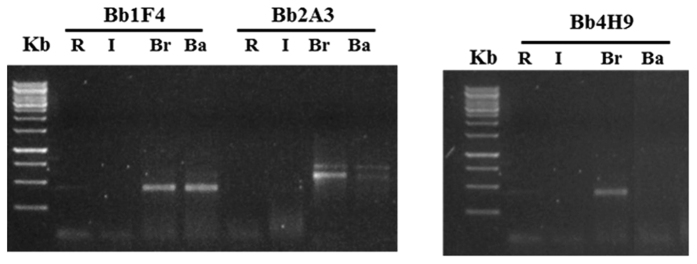
Gel electrophoretic analyses of PCR products derived from the *B. brongniartii* SSR marker amplification. The bands represent the *B. brongniartii* (Br) fungal PCR products for SSR loci Bb1F4, Bb2A3, Bb4H9 separated in 1.5% agarose. R: soil near the strawberry roots; I: soil from the inter rows; Ba. Soil inoculated with B. bassiana.

**Figure 2:**
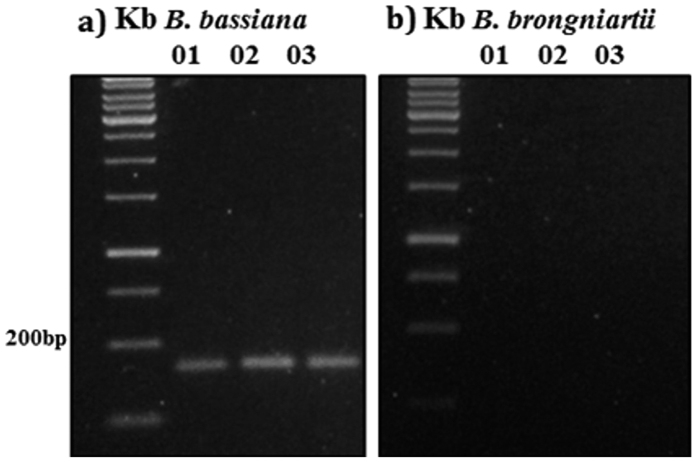
Gel electrophoretic analyses of PCR products derived from the *B. bassiana* SSR marker amplification. The bands shows the *B. bassiana* (**a**) and the *B. brongniartii* (**b**) fungal PCR product for SSR loci Ba01, Ba02, Ba03 separated in 1.5% agarose.

**Figure 3:**
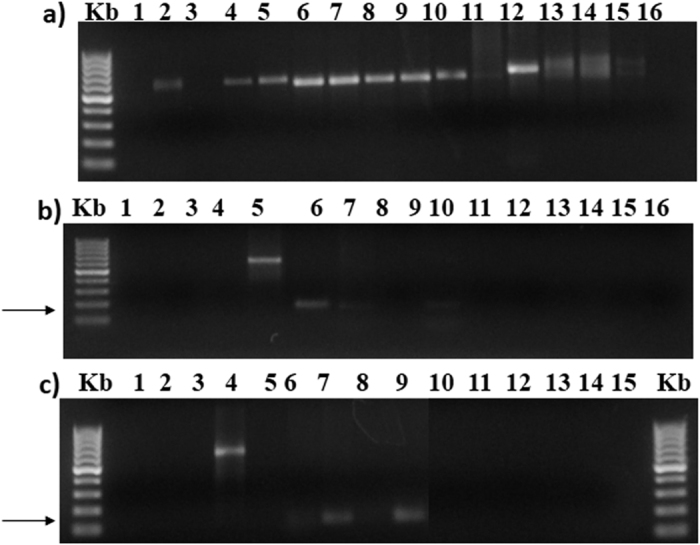
Agarose gel electrophoresis showing the PCR products amplified using the SSR primer sets from the fungal isolates listed in Table 1 . PCR products derived from (**a**) ITS1/TS4 amplification, (**b)** *Beauveria brongniartii* SSR amplification, (**c**) *B. bassiana* SSR amplification. Kb: 100 bp DNA ladder; 1: *Engyodontium album*; 2: *Eurotium rubrum*; 3: *Aspergillus tubingensis*; 4: *Penicillium pancosmium*; 5: *Beauveria* sp.; 6: *B. brongniartii* (DNA from fresh mycelium); 7: *B. bassiana* (DNA from fresh mycelium); 8: soil inoculated with *Beauveria brongniartii*; 9: soil inoculated with *B. bassiana*; 10: UPH/019; 11: UPH/018; 12: *Pochonia sp.*; 13: Soil DNA; 14: soil DNA; 15: soil DNA; 16: negative control, addition of sterile water to the PCR mix.

Three *B. bassiana* and three *B. brongniartii* SSR primer pairs were evaluated for specificity in DNA detection of our fungal strains in soil, as well as for species identification from mycelia. Only the primer Bb4H9, among those used for *B. brongniartii*, resulted capable of discriminating the fungal species, and in particular allowed a highly specific detection of *B. brongniartii* in soil (Fig. 1). Specific PCR amplification of DNA from *B. bassiana* with SSR primers was obtained for all the three considered loci. However, the primer Ba01 was selected for the following analyses because it amplified better than the other two. Amplification of the locus Bb4H9 in soil samples inoculated with *B. brongniartii* resulted in only one product, while, as expected, no amplification was obtained with the primer for the locus specific for *B. bassiana*. PCR amplification of the locus Ba01 showed only one product in soil samples inoculated with *B. bassiana* and no amplification for the locus specific for *B. brongniartii* (Fig. 2). Gel analysis revealed amplification product from *B. brongniartii* fresh mycelium (Fig. 3b), and *B. bassiana* fresh mycelium (Fig. 3c).

To further assure the specificity of these two SSR markers, the DNA of eight fungal isolates frequently present in agricultural soils or characterised by entomopathogenic ability were was amplified separately with Bb4H9 and Ba01 primers. All fungi were also amplified with ITS primers to check both their identity and the quality of extracted DNA. All the strains generated PCR products of 500 to 650 bp corresponding to the ribosomal DNA (ITS1, ITS2, and. 5.8 S rRNA) (Fig. 3a), while none of the eight strains have resulted in PCR products with neither Bb4H9 nor Ba01 primers (Fig. 3b,c). The size of the amplification product was ~200 bp and ~140 bp, from *B. brongniartii* and *B. bassiana* fresh mycelia (Fig. 3b,c).

### Sensitivity of cultivation-independent SSR detection

**Figure 4:**
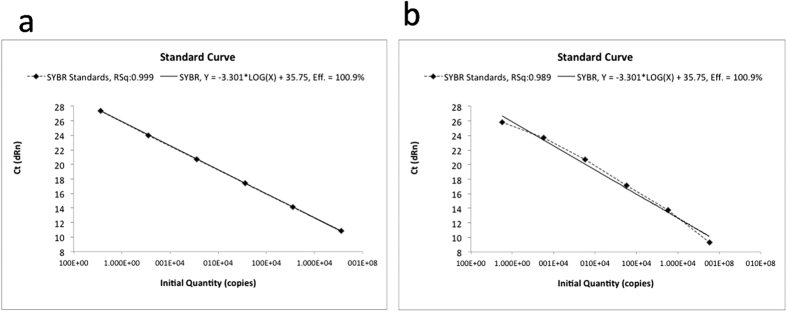
q-PCR assay: Standard curve established between Log of DNA concentration vs. cycle threshold (CT) obtained in Sybr green assay. The plots represents serially diluted (1:10) soil genomic DNA inoculated with 26.5·10^4^ and 27.25·10^4^ spores ·ml^−1^ for *B. brongniartii* (**a**) and *B. bassiana* (**b**), respectively.

The sensitivity for the discriminant detection of *B. brongniartii* and *B. bassiana* in bulk soil DNA was tested using 50 ng of bulk soil DNA extract from *Beauveria*-free samples (control) untreated or singly spiked with different quantities of genomic DNA of each fungal species. The locus Bb4H9 has been reported to be highly polymorphic, while Bb1F4, and Bb2A3 were not. The absence of any amplification products in the spiking experiments with *B. bassiana* (as reported above) further confirmed the specificity of the primers. The detection sensitivities of markers were adjusted according to the total amount of genomic DNA extracted from soil samples, and considering the relative C_t_ values (Fig. 4).

**Figure 5:**
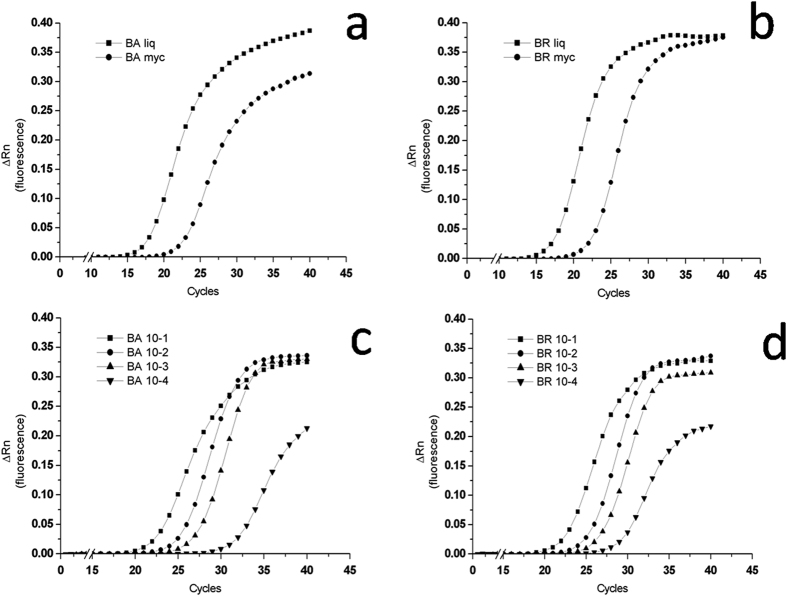
On the top, plots of PCR cycle number against PCR product amount obtained from the liquid fungal spores’ suspensions (**liq**) and the mycelium grown on solid agar (**myc**) for (**a**) *B. bassiana* and (**b**) *B. brongniartii* respectively. On the bottom, plots of PCR cycle number against PCR product amount obtained from *in vitro* soil dilutions of spores. The highest yields in the two series of samples, corresponding to the 10^−1^ dilution of the initial suspensions, were: 26.5 · 10^3^ and 27.25 · 10^3^ spores for respectively (**c**) *B. bassiana* and (**d**) *B. brongniartii*; the other curves in the plots correspond to three further ten-fold sequential dilutions of the fungal spores’ suspension (from 10^−2^ to 10^−4^).

The qPCR was highly specific when tested with the two loci on fungal DNA from the fungal pure cultures of both *B. brongniartii* and *B. bassiana* strains (Fig. 5). The qPCR results obtained from the fungal spores’ suspensions and the mycelium grown on solid agar are showed in Fig. 5a,b for *B. bassiana* and *B. brongniartii,* respectively. The results obtained from *in vitro* soil dilutions of *B. bassiana* and *B. brongniartii* spores from the qPCR are shown in Fig. 5c,d, respectively. The relation between the number of PCR cycles and the amount of PCR product obtained was similar for both *Beauveria* species (Fig. 5). The reduction in spores’ concentrations obtained with the first three dilutions (10^−1^, 10^−2^ and 10^−3^) resulted in a decreasing amount of DNA template for both fungal species, thus requiring more amplification cycles for the fluorescence signal to rise above background in the most diluted samples. After 30 cycles the fluorescence signal of the first three dilutions (10^−1^, 10^−2^ and 10^−3^) raised at comparable intensities in both fungi. The last dilution (10^−4^) resulted in a qPCR product amount that plotted separately from the others, with a very delayed Ct, confirming that a small amount of template was present at the start of the reaction (Fig. 5c,d). Moreover, more amplification cycles were required for both fungal species with the 10^−4^ dilution samples for the fluorescence signal to rise above background, resulting also in a plateau of the curve with intensities sensibly lower than those attained by the other dilutions. These data indicate that the method allows to detect a very small number of gene copies per g of soil (Table 2).

### Presence and yield of *B. brongniartii* and *B. bassiana* in the treated soils

When applied to the field situation, namely the detection and quantification of the entomopathogenic fungi *B. brongniartii* and *B. bassiana* in previously inoculated soils under field conditions, the qPCR analysis showed very low values (Table 2). Despite the very low numbers of gene copies obtained from all the trials (with *B. brongniartii* close to zero in all the trials), the data were highly repeatable and able to show statistic differences between the agronomical treatments, as in Trial A (Strawberry field) where soil samples from the root zone (Root-BA) showed a higher amount of *B. bassiana* than in the untreated soil (Root). The results however did not indicate the persistence of the fungi in the inoculated soils. In general, the *B. bassiana* gene copy values were higher than those of *B. brongniartii* in all the treatments.

**Figure 6:**
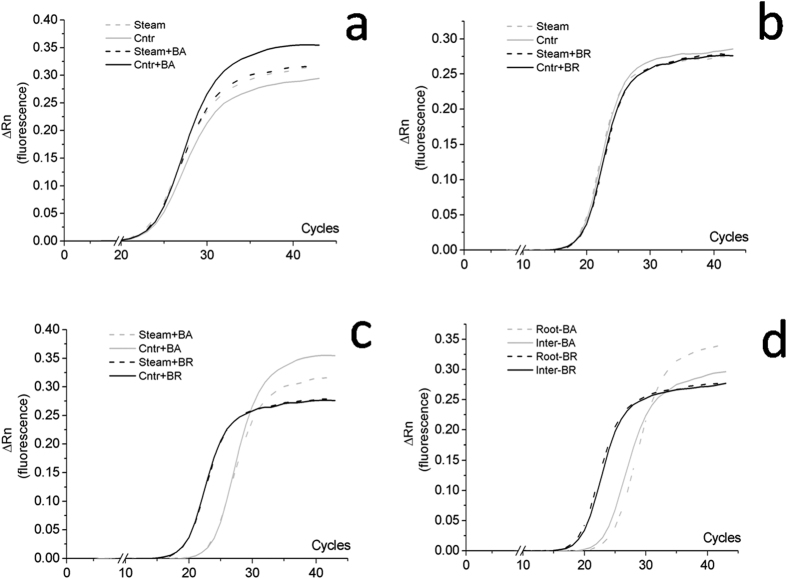
Plots of PCR cycle number against PCR product amount obtained in the trials B (plots **a–c**) and A (plot d), for the two fungal inoculants. Plot (**a**) soil inoculated with *B. bassiana* previously treated (Steam + BA) or not treated (Steam) with active steam, compared with the relevant controls (Cntr and Cntr + BA). Plot (**b**) soil inoculated with *B. brongniartii* previously treated (Steam + BR) or not treated (Steam) with active steam, compared with the relevant controls (Cntr and Cntr + BR). Plot (**c**) soil co-inoculated with *B. bassiana* and *B. brongniartii* and previously treated, or not, with active steam. Plot (**d**) soil inoculated with *B. bassiana* or *B. brongniartii* sampled near the strawberry roots (R) and from the inter rows (I).

The relation between PCR cycle number and the amount of PCR product obtained in the trials A and B, for the two fungal inoculants, is presented in Fig. 6.

In particular, in Fig. 6a raw results obtained from soil samples inoculated with *B. bassiana* and previously treated (Steam + BA) or not treated (Cntr + BA) with active steam were compared with the relevant not inoculated control samples (Steam and Cntr) consisting of not inoculated soil samples. qPCR using SSR marker as primer allowed to detect and quantify the inoculated *B. bassiana* in the soil previously disinfected or not with active steam (Fig. 6a), showing that these differences were not significant when reported as the average copy number/gram dry soil (Table 2).

In Fig. 6b are reported the results obtained from soil samples inoculated with *B. brongniartii* and previously treated (Steam + BR) or not treated (Cntr + BR) with active steam, compared with the relevant controls (Steam and Cntr). The proposed method applied to the soil samples collected from the field plots where *B. brongniartii* was applied showed that this fungus was not present or in an amount lower than the detection limit of the method (Fig. 6b), and with an amplification plateau due to lack of DNA template lower than that of *B. bassiana* (Fig. 6c).

In Fig. 6d results obtained from soil samples inoculated with *B. bassiana* or *B. brongniartii* and taken from the strawberry plants’ roots (Roots) and from the plants’ inter rows (Inter) are compared. The results from the analysis of soil samples deriving from plots co-inoculated with the two fungi showed the persistence of *B. bassiana* in those collected from the root zone (Roots-BA) and not in the samples taken from inter rows (Inter-BA), even after a significant number of PCR cycles (Fig. 6d). *B. brongniartii* was not detected in any sample irrespective of the collection sites.

## Discussion

In our study, we have coupled highly discriminant species-specific SSR primers with a real-time qPCR using the same SSR markers as primers. Such an approach allowed us to define: a) the primers that were able to detect *B. brongniartii* and *B. bassiana* in soil samples, b) that the technique could be used to monitor with sensitivity and specificity their persistence in soil, c) that the qPCR based on SSR could be used to quantify single fungal species biomass in soil.

Microsatellite markers^22^ showed a good discrimination power for filamentous fungi, and over the last few years various studies have been carried out to develop SSR markers in *Beauveria* spp.^22^,^29^,^30^. Such approach allowed to reveal fungus genetic diversity for a given species or genera, and more generally made possible the cultivation-independent detection of fungal strains in soil samples^29^,^30^. The polymorphic character of SSRs is highly discriminating in fungi, more than other DNA loci (i.e. ITS sequences or functional genes), and may represent a promising option for culture-independent detection and identification of fungal strains in environmental samples, especially if coupled with cloning and sequencing.

Specificity for a target organism is a critical element in the design or use of any PCR and qPCR real time assay. Species specificity was demonstrated in our study by the discriminant presence or absence of SSR amplification products from the two fungi using specific primers (Bb4H9 for *B. brongniartii* and Ba01 for *B. bassiana*). However, some of the tested SSR primer pairs (Bb1F4 and Bb2A3) detected both species (Figs 1 and 2). Therefore, detection specificity of SSR primers needs to be validated case by case prior to being applied to complex soil DNA samples^29^.

Protocol efficacy was firstly tested with *in vitro* fungal spores’ dilutions in soil samples, thus performing a sort of calibration and validation of the method using microcosms where a known amount of fungal reproductive units was purposely added before the DNA extraction. We assumed that if a large amount of template DNA was present at the start of the reaction, relatively few amplification cycles would be required to accumulate enough product to give a fluorescence signal above background. A higher amount of DNA template was present in the DNA extracts obtained from spores’ suspensions (liq) than in mycelia (myc) harvested from solid cultures for both *Beauveria* species. In *B. bassiana* the fluorescence signal obtained from the mycelia DNA never reached the level obtained with the spores DNA template. In *B. brongniartii* the DNA template from mycelia at the start of the reaction was lower, but at the end of the PCR cycles the fluorescence signal obtained was comparable to that obtained from spores’ template. This passage proved to be determinant for defining the threshold of fungal detection as well as for evaluating the presence of interferences in DNA recovery and amplification, due to soil components. The results obtained from microcosms and pure fungal cultures have been confirmed when analysing soil samples gathered from field experimental trials. The presence of a number of gene copies different from zero in the non-inoculated treatments, in the case of both *B. bassiana* and *B. brongniartii* plots could indicate the presence of a local, natural, population of the fungus. Being the soils naturally infested by *M. melolontha* it is reasonable that its natural parasites are also present. It is worth of notice that in both the B experiments without the steam treatment, the control showed a significant higher presence of the fungus respect to the inoculated trials, which could indicate that the added inoculums, although too low to establish in soil, had the effect of depressing the native strains of *Beauveria*.

Schwarzenbach *et al.*^29^ reported for the first time the specificity of SSR primers within bulk soil. In this work we are reporting for the first time the SSR species specificity also in soil close to the plants root system, where a higher microbial diversity is usually present, thus being theoretically a more difficult environment to trace single species. To our best knowledge, this is also the first report where this double approach (SSR and real time qPCR) has been applied for the simultaneous detection and traceability of inoculants in soil.

The marketing of microorganisms as soil inoculants for biocontrol or biofertilization requires risk assessment for regulatory and technical purposes^5^. The registration of a microbial-based product obliges the producer to present several data about the efficacy and safety of the inoculum to the competent authorities. Both efficacy and safety are related with the methods of application of the product, which can affect the viability of the inoculant as well as its establishment in the soil. Indeed, recovery of a single inoculated strain in soil microcosms for a plant growth promoting bacteria was limited to 30–40 days after inoculation^38^ and changing the sprayer technical parameters deeply affected the viability of the strain applied^39^. The method allowed to assess not only the effect of the treatment in association with other agronomical practices, but also to point out the need of increasing the dose of the inoculum, particularly of *B. brongniartii*, or to change its application method, which could explain the limited biocontrol activity recorded in the trial^40^.

Furthermore, farmers like to utilize “multifunctional” products and manufacturers prefer to market products with several activities (nutritional and plant strengtheners) because they are more likely to have effects and attract users. It appear thus evident that there is a perceived and real need for accurate and standard methods, to support the registration process and to fine tune the bio-product application strategy, suitable to monitor the persistence of the different inoculated strains and to evaluate the success of inoculation. Detection of microorganisms released in the environment with molecular methods based on PCR techniques that use natural genome polymorphism, such as the one described in this report, can largely facilitate and allow the discrimination at the strain level of natural and introduced organisms, increasing the safety of bio-inoculants for the environment and the time and economic efforts of farmers^41^,^42^,^43^.

## Conclusions

Cultivation-independent multilocus genotyping, combined with the qPCR (qPCR-SSR analysis), allowed detecting with high specificity and sensitivity the fungal inoculants even when present in a very low concentration in soil. It was possible to trace and quantify *B. bassiana* in soil after field inoculation. The lack of detection of *B. brongniartii* after the application of the bio-inoculum under field conditions showed the potentiality of the method to evaluate the efficacy of biocontrol treatments to soil. The results suggest that it represents an attractive method for reliable and flexible, as well as cost- and time-effective, detection and dosing of inoculants in soil.

## Additional Information

**How to cite this article**: Canfora, L. *et al.* Development of a method for detection and quantification of *B. brongniartii* and *B. bassiana* in soil. *Sci. Rep.* **6**, 22933; doi: 10.1038/srep22933 (2016).

## Figures and Tables

**Table 1:** List of fungal strains used in this study to test specificity of SSR marker.

Species	Isolate	Id
*Engyodontium album* (Limber) de Hoog	From soil	KU686684
*Aspergillus tubingensis* Mosseray	From saline soil	KU686682
*Eurotium rubrum* Jos. König, E. Spieckermann & W. Bremer	From saline soil	KU686683
*Stilbella* Lindau	From forest soil	KU686685
*Penicillium pancosmium* Houbraken, Frisvad & Samson	From forest soil	KU686681
*Engyodontium arenarum* (Cavara) W. Gams	From spider	KU686686
*Beauveria Beauveria Vuill.*	From soil	–
*Pochonia Bat. & O.M. Fonseca*	From adult butterfly	KU686687

**Table 2:** Data obtained in PCR amplification of target DNA from the two fungi in soil samples of trial “A” from the plants’ roots (Root) or from inter-rows soil (Inter), and in soil samples of trial “B” where two treatments were considered: soil treated with active steam (Steam) or not treated/control (Cntr).

Trial A (Strawberry field)			
*B. bassiana*		*B. brogniartii*	
Gene copy number g^−1^dry soil		Gene copy number g^−1^ dry soil	
Inter-BA	21.64 ± 10.86^a^	Inter-BR	0.00 ± 0.00^b^
Inter-cntr	6.35 ± 2.96^b^	Inter-cntr	0.01 ± 0.00^a^
Root-BA	13.84 ± 6.95^ab^	Root-BR	0.00 ± 0.00^b^
Root-cntr	11.30 ± 6.64^ab^	Root-cntr	0.01 ± 0.01^a^
Trial B (Bare field)			
Cntr	26.37 ± 10.88^a^	Cntr	0.03 ± 0.01^a^
Cntr + BA	20.06 ± 1.47^a^	Cntr + BR	0.00 ± 0.00^b^
Steam	30.23 ± 10.57^a^	Steam	0.03 ± 0.01^a^
Steam + BA	22.80 ± 3.74^a^	Steam + BR	0.00 ± 0.00^b^

Detection of genotype-specific alleles of *B. brongniartii* (BA) and *B. bassiana* (BR) in samples of soil treated with 50 ng·g^−1^ (dry soil) of *B. brongniartii* and *B. bassiana* inocula. Means of 4 laboratory replicates ± SDfrom one-way ANOVA of real time quantification data (Average copy number/gram dry soil). Different letters indicate a significant difference (p < 0.05) after Fisher test.
